# Prevalence of dry eye in Brazil: Home survey reveals differences in urban and rural regions

**DOI:** 10.1016/j.clinsp.2025.100578

**Published:** 2025-01-28

**Authors:** Leidiane Adriano Pereira, Laura Braga Arantes, Etiene Lorriane de Souza Persona, Denny Marcos Garcia, Isvander Gustavo de Souza Persona, Regina Celia Nucci Pontelli, Eduardo M. Rocha

**Affiliations:** Department of Ophthalmology, Otorhinolaryngology, and Head & Neck Surgery, Faculdade de Medicina de Ribeirão Preto, Universidade de São Paulo, Ribeirão Preto, SP, Brazil

**Keywords:** Dry eye, Prevalence, Population survey, Risk Factors, Brazil

## Abstract

**Purpose:**

A random home visit search investigated the prevalence and predictors of Dry Eye (DE) symptoms in Brazil.

**Methods:**

Through home visits to 420 urban and 180 rural residences in the countryside of Southeast Brazil, the Dry Eye Disease Short Questionnaire (DEDSQ) was applied to volunteers 40 years and older. The predictive value of the DEDSQ and the risk factors were investigated.

**Results:**

DE is more frequent in the urban population (38.1 % vs. 20 %; *p* < 0.0001) and was higher in females (37.5 % and 21.5 %; *p* < 0.0001). In the urban area, DE is associated with dyslipidemia (*p* < 0.0001), visual display terminal use > 2 h/day (*p* < 0.0001), and pterygium (*p* < 0.001), and in the rural area with dyslipidemia (*p* < 0.01) and prior ocular surgery (*p* < 0.05). In women, the predictors are antiallergics, chronic pelvic pain, fibromyalgia, and visual display terminal use > 2h/day. Aging was associated with distinct risk factors. DEDSQ showed 78.5 % of sensitivity and 71.1 % specificity.

**Conclusion:**

This first domiciliary study in Brazil reveals that DE symptoms were more prevalent in the urban area and among women. DE has distinct risk factors and a high prevalence in Brazil.

## Highlights

• Dry eye is more frequent in the urban than in the rural population.
• Dry eye is associated with dyslipidemia, visual display terminal, and ocular surface problems.
• Dry eye is more frequent in women and the associated factors are associated with antiallergics, chronic pelvic pain, and fibromyalgia.
• Dry eye in the elderly has distinct clinical associated factors.

## Introduction

Dry Eye (DE) is a growing public health problem and one of the most frequent reasons for seeking eye care.^1^ The definition of DE, published in 2017 takes into account the multifactoriality of this pathology, the changes in the tear film, and the symptoms suffered by the patients. Diagnosis and epidemiology are still challenges in DE.^2^^,^^3^

The prevalence of DE varies a lot around the World, and the reasons are associated with geographic, demographic, genetic background, environmental, method of evaluation, and other factors.^3^

Previous studies applied symptom questionnaires in the general population and demonstrated a DE prevalence from 4.3 % to 14.5 % in the USA,^4^, ^5^, ^6^, ^7^ 32.1 %‒45.1 % in Saudi Arabia,^8^, ^9^, ^10^, ^11^ and in China, it ranged from 21 % to 51.1 %.^12^, ^13^, ^14^ Therefore, DE represents a common cause for seeking eye care, it is associated with several comorbidities, and the incidence is growing.^15^^,^^16^

An epidemiological study conducted in Brazil investigated the spontaneous demand for ophthalmologist's offices for Dry Eye Disease Symptoms (DEDS), revealing a prevalence of 12.8 % in this population.^17^ One other study in a metropolitan area in southeastern Brazil (Vitoria, ES) found DEDS in 18 % of the sample.^18^ In another study, in Brazil, with university students, 34.4 % presented OSDI scores higher than 22, DE was more frequent among women, contact lens users, and those with video display exposure higher than 6h/day.^19^ Recently, a study in the metropolitan area of Sao Paulo city among 582 adults 18 years or older, interviewed by phone call applying the DEDS Short Questionnaire (DEDSQ) revealed a prevalence of DE of 24.4 % and higher association with female sex, aging, and systemic hypertension and eye drops use.^20^

As part of the present project, the authors evaluated the frequency of pterygium in the population of the southeast countryside of Brazil and observed a total frequency of 21 %, a higher frequency in men and similar among urban and rural adult populations. Moreover, the authors observed a higher frequency of ocular surface fluorescein staining in those individuals, suggesting an association with DE.^21^

Determining the prevalence and risk factors of DE is relevant to predicting the events related to corneal disease, as observed in a longitudinal study in Taiwan, where the rate of DE was estimated at 7.85 % and its association with corneal damage was higher among younger individuals, women, and people with Diabetes Mellitus (DM) and Rheumatoid Arthritis (AR), along of 15 years of observation.^22^ The mechanisms of the association between DED and corneal disease were recently revised.^23^

This study investigated the DE prevalence involving two adult populations (rural and urban) in Brazil's southeastern countryside, following the active household search method, and the predictive value of the DEDSQ and the DE risk factors.

## Materials and methods

### Type of study and sample

After the approval by the Committee of Ethics (CAAE n° 53,174,416.8.0000.5440), the authors conducted a cross-sectional, population-based study in two cities: Ribeirão Preto (21°10′40″S, 47°48′36″W) and Cássia dos Coqueiros (21°16′58″S, 47°10′12″W), both of São Paulo State, Southeast of Brazil.

The population of Ribeirão Preto, São Paulo, Brazil, is 698.259 inhabitants, with 124,510 women and 101,282 men aged 40 and over residing in this town. It is one of the largest cities in São Paulo State, with industries, hospitals, universities, a regional airport, and other characteristics of a medium-sized town, according to the Brazilian Institute of Geography and Statistics (IBGE).^24^ The population of Cássia dos Coqueiros, São Paulo, Brazil, is 2799 inhabitants, 1287 women, and 1347 men; the inhabitants of 40 years or more are 1099, of which 567 are men, and 532 are women. Cássia dos Coqueiros is a small village, where half the population lives in a rural area, and agricultural and livestock activities represent most of the occupation and the economy (IBGE).^25^

Participants were recruited from both sexes, aged ≥40 years, 180 from a list of the Primary Health Care Centers in Cássia dos Coqueiros, and 420 participants from lists of the five Primary Health Care centers in the west region in Ribeirão Preto. They were interviewed through random home visits, without prior contact, to gather demographics, DEDS, chronic comorbidities, and daily habits and activities.

The data harvesting was done in the dry season in the southeast region of Brazil. In the rural area of Cassia dos Coqueiros, SP, the data harvest was in July 2016, and in the urban area of Ribeirao Preto, SP, the data was harvested between July and August 2017. The temperature range and relative humidity rates were similar.

A random sample was invited to a complimentary eye exam, under the following criteria: all participants with positive DEDSQ and 1 in 5 participants with negative DEDSQ.

### DEDSQ positivity criteria and risk factors evaluation

The authors used the DEDSQ symptom questionnaire translated and validated in Portuguese in 2017.^26^ The answers “often” or “always” to questions 01 (Do you feel your eyes dry?) and 02 (Do you feel your eyes irritated?) or the “yes” answer to question 3 (Have you ever had a dry eye diagnosis?) classified the questionnaire as positive for DE.

Based on previous studies,^3^, ^4^, ^5^, ^6^, ^7^, ^8^, ^9^, ^10^, ^11^, ^12^, ^13^, ^14^ the following risk factors were questioned as “yes or no” during the home interviews and DEDSQ application: Diabetes Mellitus, Menopause (for women), Rheumatic diseases, Hanseniasis, Trachoma, Chemotherapy and radiotherapy, Ocular Surgery, Contact lenses wearing, Thyroid diseases, digital electronic screen daily use longer than 2 h, antidepressant medication in the last 30 days, antiallergic medication in the last 30 days, chronic pelvic pain for longer than 3 months, fibromyalgia, dyslipidemia, and pterygium. Their frequency was calculated and correlated with the positivity for DE in the DEDSQ.

### DE clinical confirmation

DE confirmation in individuals examined clinically took into account the criteria previously published and took into consideration the score in the Ocular Surface Disease Index (OSDI) questionnaire and the ocular surface thresholds indicated (Table 1).^27^ The same researcher (L.A.P.) performed all measurements under similar test conditions.

**Table 1:** Exam Parameters to indicate DE applied in the random volunteers recruited among individuals positive and negative for DEDSQ from both areas, Cassia dos Coqueiros, SP (2016) and Ribeirão Preto, SP (2017).^27^

**Test**	**Value**
Schirmer Test	≤ 10 mm
TFBUT	< 10 s
CFS	score ≥ 3
LGCS	score ≥ 3
OSDI	score > 13

DE, Dry Eye; TFBUT, Tear Film Break Up-Time; CFS, Corneal Fluorescein Staining; LGCS, Lissamine Green Conjunctival Staining; OSDI, Ocular Surface Disease Index.

The authors quantified the Ocular Surface Disease Index (OSDI) with an OSDI Portuguese-validated version of the questionnaire.^28^

Schirmer test measured the tear flow using a strip of filter paper (Eye Pharma, SP, Brazil) and observed the wetting of the tape after 5 min on the lower eyelid without anesthesia. The authors quantified Tear Film Breakup Time (TFBUT) and Corneal Fluorescein Staining (CFS) by instilling a drop of 1 % sodium fluorescein eye drops (Ophthalmos Ltd., SP, Brazil). First, the authors checked the TFBUT and later, we quantified the CFS following the 15-point National Eye Institute (NEI) scale.

The authors evaluated the Lissamine Green Conjunctival Staining (LGCS) with a strip of Lissamine green paper (Eye Pharma, SP, Brazil) as described by the van Bijsterveld scale.

Clinical exams and OSDI questionnaires were applied to the random sample of volunteers a few days after the house visits in their respective local healthcare centers. In the rural area of Cassia dos Coqueiros, SP, the clinical data was harvested in July 2016, and in the urban area of Ribeirao Preto, SP, the data was harvested in August 2017. The OSDI questionnaire was applied minutes before the clinical exam.

### Statistical analysis

For statistical calculations, the authors used GraphPad Prism software version 8.3.1 (GraphPad Software, San Diego, California, USA) and R 3.6.0 (R Foundation for Statistical Computing, Vienna, Austria).

The total sample was determined by the simple random sampling formula: *n* = Z^2^ [P(1-P)]/D^2^, where Z is a constant equal to 1.96, n is the sample size, P is the expected prevalence, here assumed as 10 %, based on previous studies, and D is the maximum acceptable error, adopted as 2.5 % in the estimate. The calculation resulted in approximately 600 participants.

The authors used the Fisher exact test for categorical analysis and the Mann-Whitney U test for comparison between continuous data from the groups. The authors calculated logistic regression analysis to determine the correlation of DEDSQ positivity and a set of predictor variables in both subgroups (urban and rural) and also combined. The authors estimated DEDSQ sensitivity and specificity. The adopted p-value was < 0.05.

## Results

### Demography

A total of 600 household participants, 419 women (69.8 %) and 181 men (30.2 %), were interviewed. The participants' ages ranged from 40 to 94 years (mean ± standard deviation was 62.7 ± 12.4 years).

The frequency of positive DEDSQ was higher in females than in males (37.5 % and 21.5 %, respectively; *p* < 0.0001), and the prevalence was higher in the urban than in the rural area (38.1 % and 20 %, respectively; *p* < 0.0001).

Positive DEDSQ according to the participant's age group showed that the frequency variates from 20.4 % to 40 % according to the advance to older age (*p* < 0.018). However, aging as a risk factor is significant only in the urban area (*p* < 0.001) (Table 2). There was a lower association for dry eye in the male sex group compared to females in the urban area (29.1 vs. 54.9 %, *p* < 0.001), but this risk factor, identified by DEDSQ, is not present in the rural area (17 % vs. 21.1 % for males and females respectively, *p* = 0.3) (Table 2).

**Table 2:** Prevalence of positive dry eye symptoms questionnaires, according to sex and age group, spread over decades among the 600 participants of the study of dry eye epidemiology in Brazil (Fisher exact test).

**Characteristic**	**Frequency of positive DESDQ (%)**	**Overall (*n* = 600)**			**Urban (*n* = 480)**			**Rural (*n* = 120)**		
		**OR**	**95 % CI**	**p-value**	**OR**	**95 % CI**	**p-value**	**OR**	**95 % CI**	**p-value**
**Sex**				<0.001			<0.001			0.3
F	37.5	‒	—		‒	‒		‒	‒	
M	21.5	0.44	0.29, 0.66		0.40	0.25, 0.63		0.61	0.22, 1.56	
**Age group**				0.018			<0.001			0.8
40‒49	21 (20.4)	‒	‒		‒	‒		a	a	
50‒59	42 (29.4)	1.78	0.98, 3.30		1.71	0.92, 3.24		‒	‒	
60‒69	65 (38.9)	2.70	1.53, 4.90		3.57	1.97, 6.68		0.47	0.14, 1.68	
70‒79	45 (36)	2.18	1.20, 4.07		2.64	1.39, 5.10		0.53	0.15, 1.93	
80‒89	21 (36.8)	2.54	1.22, 5.32		3.63	1.60, 8.29		0.45	0.09, 2.00	
90+	2(40)	2.19	0.28, 14.1		3.25	0.12, 84.5		0.72	0.03, 9.38	

n, Number of participants; CI, Confidence Interval; DEDSQ, Dry eye Disease Symptoms Questionnaire; OR, Odds Ratio.

a In the rural area, no individuals aged between 40 and 49 were observed.

### Risk factors for deds

In the urban area, ten risk factors showed a positive correlation with DE, in the univariate analysis: rheumatological diseases (*p* = 0.0002), thyroid diseases (*p* = 0.003), chronic use of antidepressants (*p* = 0.0001), chronic use of antiallergics (*p* < 0.0001), chronic pelvic pain (*p* = 0.0004), fibromyalgia (*p* = 0.0014), dyslipidemia (*p* < 0.0001), eye surgery (*p* = 0.01), visual display terminal > 2h/day (*p* < 0.0001), and pterygium (*p* = 0.0013). In multivariate analysis, three predictors showed this 0.0001), eye surgery (*p* = correlation: dyslipidemia; *p* < 0.0001), visual display terminal use > 2h/day (*p* < 0.0001), and pterygium (*p* = 0.0007) (Table 3).

**Table 3:** Correlation between the positivity of the dry eye symptoms questionnaire and the systemic and ocular risk factors in the total sample (*n* = 600), urban (*n* = 420), and rural area (*n* = 180) of the study of dry eye in Brazil.

Risk factors	Positive DEDSQ			OR (95 %CI)^a^		
	Sample			Sample		
	Total	Urban	Rural	Total	Urban	Rural
Systemic						
Diabetes mellitus	60	50	10	1.34 (0.91‒1.97)	1.36 (0.89‒2.12)	1.20 (0.53‒2.69)
Postmenopausal women	124	98	26	1.77 (1.13‒2.82)	1.61 (0.96‒2.70)	5.45 (2.9‒24.25)
Rheumatological diseases	42	37	5	2.23 (1.42‒3.49)	2.96 (1.70‒5.18)	1.00 (0.38‒2.66)
Leprosy	3	2	1	6.22 (0.92‒80.92)	3.28 (0.38‒47.67)	Infinite (0.43‒∞)
Chemotherapy	7	7	‒	0.84 (0.32‒1.95)	0.80 (0.31‒2.04)	0 (0‒4.64)
Radiotherapy	9	7	2	1.25 (0.51‒2.77)	0.80 (0.31‒2.04)	8.41 (0.94‒122.5)
Thyroid diseases	36	29	7	1.59 (0.99‒2.56)	2.52 (1.38‒4.58)	0.96 (0.37‒2.27)
Antidepressants	64	53	11	2.03 (1.36‒2.99)	2.5 (1.56‒3.95)	1.37 (0.63‒3.02)
Antiallergics	34	32	2	3.05 (1.79‒5.13)	3.81 (2‒7.32)	0.79 (0.17‒3.12)
Chronic pelvic pain	16	13	3	4.40 (1.95‒9.89)	7.58 (2.16‒25.22)	2.53 (0.64‒10.26)
Fibromyalgia	24	20	4	2.83 (1.53‒5.16)	3.57 (1.69‒7.51)	1.87 (0.6‒6.59)
Dyslipidemia	96	75	21	3.12 (2.19‒4.49)	4.22 (2.67‒6.54)	2.63 (1.29‒5.72)
Ocular						
Eye Surgery	67	52	15	2.10 (1.43‒3.08)	1.80 (1.15‒2.79)	3.4 (1.60‒7.66)
VDT > 2h/day	42	40	2	3.17 (1.91‒5.23)	7.54 (3.85‒15.20)	0.34 (0.08‒1.46)
Contact lens	4	4	‒	2.78 (0.62‒12.57)	2.2 (0.58‒8.79)	‒
Trachoma	5	5	‒	5.26 (1.12‒26.58)	4.16 (0.88‒21.06)	‒
Pterygium	55	47	8	1.93 (1.27‒2.89)	2.22 (1.39‒3.60)	1.24 (0.52‒2.85)
Multivariate analysis						
	OR (95 %CI)^b^					
Risk factors	Total	Urban	Rural			
Postmenopausal women	1.94 (1.28‒2.97)	‒	1.92 (0.84‒4.63)			
Rheumatological diseases	1.23 (0.71‒ 2.12)	1.89 (0.98‒3.69)	‒			
Thyroid diseases	‒	1.61 (0.79‒3.29)	‒			
Antidepressants	1.18 (0.75‒ 1.85)	1.45 (0.82‒2.56)	‒			
Antiallergics	1.69 (0.89‒ 3.19)	2.08 (0.99‒4.44)	‒			
Chronic pelvic pain	3.17 (1.19‒8.98)	3.27 (0.83‒6.78)	‒			
Fibromyalgia	1.32 (0.60‒2.87)	1.22 (0.45‒3.32)	‒			
Dyslipidemia	2.65 (1.77‒3.99)	3.38 (2.05‒5.62)	2.30 (1.04‒5.16)			
Eye Surgery	1.96 (1.28‒3.01)	1.62 (0.96‒2.72)	3.35 (1.47‒ 7.63)			
VDT > 2 h/day	3.78 (2.14‒6.73)	6.59 (3.14‒14.75)	‒			
Trachoma	3.53 (0.69‒26.18)	‒	‒			
Pterygium	2.30 (1.45‒3.66)	2.61 (1.50‒4.57)	‒			

n, Number of participants; CI, Confidence Interval; DEDSQ, Dry Eye Disease Symptoms Questionnaire; VDT, Visual Display Terminal.

a Fisher's exact test. ^b^ Logistic regression analysis. Note: The authors excluded from the multivariate analysis risk factors without statistical significance in the univariate analysis.

In the rural area, three risk factors presented a positive correlation with DED in univariate analysis: postmenopausal women (*p* = 0.01), dyslipidemia (*p* = 0.013), and eye surgery (*p* = 0.003). In multivariate analysis, two predictors showed this correlation: dyslipidemia (*p* = 0.004) and eye surgery (*p* = 0.04) (Table 3).

Considering the combined rural and urban sample of participants, eleven predictors presented a positive correlation with DEDS: postmenopausal women (*p* = 0.02), rheumatic diseases (*p* = 0.0008), chronic use of antidepressants (*p* = 0.0005), chronic use of antiallergics (*p* < 0.0001), chronic pelvic pain (*p* = 0.0006), fibromyalgia (*p* = 0.0012), dyslipidemia DEDSQ (*p* < 0.0001), past ocular surgery (*p* = 0.0002), visual display terminal use > 2h/day (*p* < 0.0001), trachoma (*p* = 0.04), and pterygium (*p* = 0.002). In multivariate analysis, six risk factors showed this correlation: postmenopausal women (*p* = 0.002), chronic pelvic pain (*p* = 0.02), dyslipidemia (*p* < 0.002), a visual display terminal use > 2h/day (*p* < 0.0001), and pterygium (*p* = 0.0004) (Table 2).

The impact of systemic and ocular predictors on the likelihood of a positive DEDSQ according to the sex and the age of participants in the total sample found that the female sex interfered in the DEDSQ positivity in the following risk factors: chronic use of antiallergic (*p* = 0.0002), chronic pelvic pain (*p* = 0.0006), fibromyalgia (*p* = 0.0184), and visual display terminal use > 2h/day (*p* < 0.0001), and male sex did not present any particular distinct factor (Table 4).

**Table 4:** Impact of Systemic and Ocular Predictors on the Likelihood of a Positive Dry Eye Disease Symptoms Questionnaire results according to the sex of participants in the epidemiology study of dry eye in Brazil.

**Risk factors**	**Positive DEDSQ**		**OR (95 %CI)**^a^	
	**Women** **(*n* = 419)**	**Men** **(*n* = 181)**	**Women**	**Men**
**Systemic**				
Rheumatological diseases	38	4	1.72 (1.06‒2.78)	5.29 (1.35‒21.51)
Antidepressants	56	8	1.58 (1.03‒2.43)	3.41 (1.21‒8.6)
Antiallergics	32	2	3.27 (1.8‒6.14)	1.04 (0.21‒5.06)
**Chronic pelvic pain**	**15**	**1**	**5.43 (1.91‒13.78)**	1.22 (0.09‒8.36)
**Fibromyalgia**	**23**	**1**	**2.19 (1.15‒4.08)**	Infinite (0.40‒∞)
Dyslipidemia	76	20	2.38 (1.57‒3.57)	6.06 (2.77‒12.62)
**Ocular**				
**Eye Surgery**	46	21	1.6 (1‒2.53)	5.2 (2.47‒11.39)
**VDT > 2h/day**	**35**	**7**	**3.29 (1.81‒5.82)**	2.60 (0.99‒7.54)
Trachoma	4	1	3.4 (0.78‒17.98)	Infinite (0.40‒∞)
Pterygium	34	21	1.85 (1.12‒3.07)	3.71 (1.81‒7.72)

n, Number of participants; CI, Confidence interval; DEDSQ, Dry Eye Disease Symptoms Questionnaire.

a Fisher's exact test. Note: The authors excluded from the adjusted analysis by sex risk factors without statistical significance in the univariate analysis.

The authors found that the age < 65 years interfered with the DEDSQ positivity in the following risk factors: antidepressants (*p* < 0.0001), chronic pelvic pain (*p* = 0.02), and eye surgery (*p* = 0.0003); and age ≥ 65 years interfered in the DEDSQ positivity in participants with rheumatological diseases (*p* = 0.0017) (Table 5).

**Table 5:** Impact of systemic and ocular predictors on the likelihood of a positive Dry Eye Disease Symptoms Questionnaire according to the age group < 65 years and ≥ 65 years in the epidemiology study of dry eye in Brazil.

**Risk factors**	**Positive DEDSQ**		**OR (95 %CI)**^a^	
	**Age < 65y** **(*n* = 336)**	**Age ≥ 65 y** **(*n* = 264)**	**Age < 65y**	**Age ≥ 65y**
Systemic				
**Rheumatological diseases**	11	31	1.43 (0.64‒3.06)	**2.67 (1.45‒4.78)**
**Antidepressants**	41	23	**3.59 (2.09‒6)**	1.02 (0.57‒1.80)
Antiallergics	18	16	3.54 (1.73‒7.39)	2.55 (1.12‒5.73)
Chronic pelvic pain	10	6	3.22 (1.19‒8.25)	Infinite (2.51‒∞)
Fibromyalgia	13	11	2.24 (1.04‒4.84)	5.21 (1.78‒15.19)
Dyslipidemia	42	54	3.06 (1.83‒5.13)	3.02 (1.8‒5.02)
**Ocular**				
**Eye Surgery**	**24**	**43**	**3.47 (1.8‒6.49)**	1.42 (0.85‒2.36)
VDT > 2h/day	32	10	3.71 (2.1‒6.7)	3.72 (1.33‒9.98)
Trachoma	0	5	‒	4.48 (0.93‒22.77)
Pterygium	26	29	1.81 (1.03‒3.19)	2.03 (1.12‒3.7)

n, Number of participants; y, years; CI, Confidence Interval; DEDSQ, Dry Eye Disease Symptoms Questionnaire.

a Fisher's exact test. The authors excluded from the adjusted analysis age risk factors without statistical significance in the univariate analysis.

### DED clinical diagnosis

The authors compared the DE clinical tests with the DEDSQ results among 128 participants from the urban area and 45 participants from the rural area. In the urban, rural, and combined groups (total sample), participants with positive DEDSQ obtained higher OSDI values than participants who presented negative DEDSQ (*p* < 0.0001 for all).

**Fig. 1:**
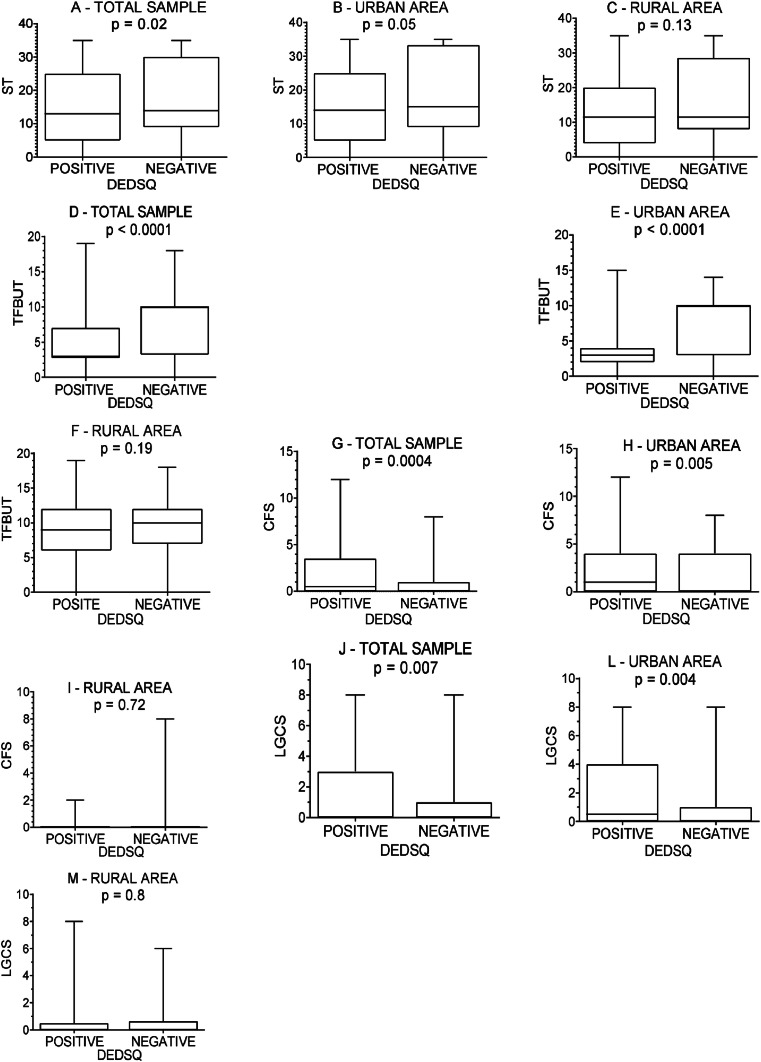
Correlation between dry eye clinical test results (ST, Schimer Test; TFBUT, Tear Film Break Up Time; CFS, Corneal Fluorescein Staining; LGCS, Lissamine Green Conjunctival Staining) and result of the Dry Eye Disease Symptoms Questionnaire (DEDSQ) in the evaluation of 173 participants of the dry eye epidemiology study in Brazil, 128 participants from the urban area, and 45 participants from the rural area. Mann-Whitney test.

In the total sample, participants with negative DEDSQ had higher values in ST than participants with positive DEDSQ (Fig. 1A), (*p* = 0.02). In the total sample and urban group, participants with negative DEDSQ had higher values in TFBUT than participants with positive DEDSQ (Fig. 1D and E, respectively), (*p* < 0.0001 for both). In the total sample and urban group, participants with positive DEDSQ had higher scores in CFS than participants with negative DEDSQ (Fig. 1G and H, respectively), *p* = 0.0004 and *p* = 0.005, respectively. In the total sample and the urban area, participants with positive DEDSQ had higher scores in LGCS than participants with negative DEDSQ (Fig. 1J and L, respectively), *p* = 0.007 and *p* = 0.004, respectively. Those mentioned agreement between DEDSQ frequency of positivity or negativity and ocular surface tests mean values indicate clinical consistency on their values.

### DEDSQ sensitivity and specificity

Analyzing the results of clinical tests (ST, TFBUT, CFS, and LGCS) and considering at least one of the positive criteria for the diagnosis of DE, the authors found 135 participants with any of these positive tests, 106 of these participants also had positive DEDSQ, revealing DEDSQ sensitivity of 78.5 % and specificity 71.1 % (AUC = 0.70). Rearranging the threshold of the diagnostic tests did not substantially change the DEDSQ sensitivity and specificity (data not shown).

## Discussion

The present work reveals the demographic profile of DE symptoms in Brazil and shows the distinction in prevalence between urban and rural areas. It also reveals the risk factors and the predictive value of DEDSQ in domiciliar interviews.

A study in Saudi Arabia also compared the prevalence of DE in urban and rural areas; the interviews were conducted in basic health centers, using a six-item symptom questionnaire for participants of both sexes, with an average age of 39.3 ± 14.1 years. The Saudi Arabian study found a higher prevalence of DE in urban than in rural areas (33.4 % and 30.5 %, respectively), but without statistical significance.^8^

DEDSQ demonstrated an easy application and adequate sensitivity and specificity for the screening of cases, possibly to be used by the general practitioner and also by the primary health care team.

DE prevalence is higher in females than in males, and presents distinct associated factors, as expected. This is in agreement with an extensive epidemiological study on DE in Brazil in 2018.^17^ Also, in Canada, a recent population survey showed a higher prevalence of DE and the female sex.^9^

Aging has been widely demonstrated as a risk factor for DED in other studies.^4^^,^^7^^,^^9^^,^^17^ Here, the authors observed that the frequency of participants with a positive DEDSQ increased with age. The authors also noted that age ≥65 years impacted the presence of DE in participants with rheumatological diseases more than in participants without this clinical condition.

The authors included in the assessment of risk factors fibromyalgia and chronic pelvic pain, considering that neuropathic pain influences the presence of DEDS and both conditions presented positive correlations and clinical association with DE symptoms in both univariate and multivariate analyses, in agreement with previous work.^29^^,^^30^ The authors observed that the female sex correlates with DEDS positivity, both in participants with fibromyalgia and in participants with chronic pelvic pain.

Diabetes mellitus did not present a correlation with DEDS, which was also observed in previous studies carried out in Brazil, Saudi Arabia, Canada, and the USA.^7^^,^^9^^,^^11^^,^^17^^,^^21^ However, in a retrospective study conducted in China in 2015 in a population-based hospital, there was a positive correlation between diabetes mellitus and DED.^31^ The heterogeneity and differences in disease time length and glycemic control contribute to this discordance among studies relating to DED and DM.

Assessing the ocular risk factors in the present study, the authors found that pterygium was associated with the symptoms of DE. A survey carried out in Indonesia in 2002, also demonstrated this positive association.^32^ However, a study showed no correlation between pterygium and DE in Canada, and this might have occurred due to the small number of patients with this factor in their study.^9^ The previous history of ocular surgery was positively related to DE in this present study, which is in agreement with the earlier epidemiological study conducted in Brazil, among outpatient individuals,^17^ and at odds with the 2016 USA study that investigated 784 participants who reported a diagnosis of DE and answered a questionnaire, demonstrating that any past eye surgery is not associated with eye surface symptoms but it is associated with vision-related DE symptoms.^33^ This difference in results can be explained by differences in the methodology.

The relationship between visual display terminal use > 2h/day, and the presence of DE was strongly demonstrated in the present study. This result also was found by a cross-sectional study in Japan, and studies in which the use of these screens for at least 6h/day also showed a positive association DE.^17^^,^^31^^,^^34^ Besides, the present study also demonstrated that the female sex is impacted by the presence of DED with visual display terminal use.

A study in Korea investigated the association between DE and metabolic outcomes in the general population and found an association between hypertriglyceridemia and DE in women.^35^ The authors observed in the present study that both women and men showed a positive association between dyslipidemia and DEDS.

Trachoma has been associated with DE for a long time and was confirmed here, possibly due to scar keratoconjunctivitis, resulting from this infection, in the present work, the small number of individuals with trachoma might affect the analysis to allow the high odds ratio.^3^^,^^36^

The prevalence of DEDS is higher in urban areas and similar to the prevalence observed in Sao Paulo City, Brazil.^21^ It was remarkable that distinct and more factors associated with DED were found in the urban compared to the rural population. Fibromyalgia and chronic pelvic pain, included here as markers of hyperalgesia and allodynia, were associated with DE in the urban area in the present and in previous works.^37^^,^^38^ Lifestyle may include habits that can explain the higher prevalence of DED in urban areas. The causes may include environmental contaminants and other factors that were recently reviewed in the Dry Eye Workshop organized by the Tear Film and Ocular Surface Society (DEWS TFOS) and published in an entire issue of The Ocular Surface Journal.^38^^,^^39^

The present work has several limitations. First, the authors did not address the whole population since the initial intention was to investigate DE in the elderly and their risk factors. In order to estimate the risk factors among the urban and rural populations and detect the causes of the higher frequency of DE in the first group, a nutritional questionnaire, indoor pollution investigation, and professional habits would be helpful. A simultaneous investigation and a correlation with local environmental conditions would also be very interesting. Those points should be addressed in future population studies to investigate DE on a population basis.

After this data harvesting, the COVID-19 pandemic revealed that both the wild spread of coronavirus in the population and the massive and prolonged use of facial masks turned risk factors for ocular surface chronic discomfort.^39^^,^^40^ An updated evaluation of the prevalence of DE in the Brazilian population is necessary to measure the impact of the pandemic.

In conclusion, the authors observed that DE symptoms in Brazil are associated with various clinical, demographic, and environmental factors, some of them already identified in other parts of the World. The prevalence of DE is high in Brazil and DEDSQ is an adequate tool for domiciliary epidemiological study. A remarkable distinction between the prevalence and associated factors comparing rural and urban populations negatively affected the urban areas.

## Ethical compliance

None of the authors is sponsored by the pharmaceutical industry or any other private source. The project was approved by the Committee of Ethics in Research of the Ribeirao Preto Medical School and Clinical Hospital of Ribeirão Preto Medical School, University of Sao Paulo, Brazil (CAAE n° 53,174,416.8.0000.5440).

## Declaration of competing interest

The authors declare no conflicts of interest.
